# Supplementary material for: Risk factors for obstructed labour in Eastern Uganda: A case control study
Source: PLoS One. 2020 Feb 10;15(2):e0228856. doi: 10.1371/journal.pone.0228856 (PMC7010384; doi:10.1371/journal.pone.0228856)
Supplement: S1 File — (DOCX) [file pone.0228856.s001.docx]

**Post hoc power calculations**

**1. Referral status, observed OR 6.8**

. power twoproportions 0.682 0.167, test (chi2) n (540) continuity

Estimated power for a two-sample proportions test

Pearson's chi-squared test; Ho: p2 = p1 versus Ha: p2! = p1

Study parameters: alpha = 0.0500, N = 540, N per group = 270, delta = -0.5150 (difference),

p1 = 0.6820, p2 = 0.1670

Estimated power: = 1.0000

**2. Use of local herbs, observed OR 2.43**

. power twoproportions 0.596 0.293, test(chi2) n(540) continuity

Estimated power for a two-sample proportions test

Pearson's chi-squared test; Ho: p2 = p1 versus Ha: p2! = p1

Study parameters: alpha = 0.0500, N = 540, N per group = 270, delta = -0.3030 (difference)

p1 = 0.5960, p2 = 0.2930

Estimated power: =1.0000

**3. History of having developed an emergency delivery, observed OR 0.56**

. power twoproportions 0.293 0.346, test (chi2) n(540) continuity

Estimated power for a two-sample proportions test

Pearson's chi-squared test; Ho: p2 = p1 versus Ha: p2! = p1

Study parameters: alpha = 0.0500, N = 540, N per group = 270, delta = 0.0530 (difference),

p1 = 0.2930, p2 = 0.3460

Estimated power: = 0.2322

**4. Number of ANC visits attended, observed OR 0.95**

. power twoproportions 0.565 0.567, test(chi2) n(540) continuity

Estimated power for a two-sample proportions test

Pearson's chi-squared test

Ho: p2 = p1 versus Ha: p2! = p1

Study parameters: alpha = 0.0500, N = 540, N per group = 270, delta = 0.0020 (difference)

p1 = 0.5650, p2 = 0.5670

Estimated power = 0.0409

**5. Prime parity, observed OR 2.15**

. power twoproportions 0.556 0.293, test(chi2) n(540) continuity

Estimated power for a two-sample proportions test

Pearson's chi-squared test; Ho: p2 = p1 versus Ha: p2! = p1

Study parameters: alpha = 0.0500, N = 540, N per group = 270, delta = -0.2630 (difference)

p1 = 0.5560, p2 = 0.2930

Estimated power: power = 1.0000

**6. Occupation of the spouse, observed OR 1.44**

. power twoproportions 0.163 0.215, test(chi2) n(540) continuity

Estimated power for a two-sample proportions test

Pearson's chi-squared test; Ho: p2 = p1 versus Ha: p2! = p1

Study parameters: alpha = 0.0500, N = 540, N per group = 270, delta = 0.0520 (difference)

p1 = 0.1630, p2 = 0.2150

Estimated power: = 0.2990

**7. Level of education of the spouse, observed OR 0.57**

. power twoproportions 0.467 0.274, test(chi2) n(540) continuity

Estimated power for a two-sample proportions test

Pearson's chi-squared test, Ho: p2 = p1 versus Ha: p2! = p1

Study parameters: alpha = 0.0500, N = 540, N per group = 270, delta = -0.1930 (difference)

p1 = 0.4670, p2 = 0.2740

Estimated power: = 0.9959

**8. Distance to the nearest health facility, observed OR 0.94**

. power twoproportions 0.241 0.182, test(chi2) n(540) continuity

Estimated power for a two-sample proportions test

Pearson's chi-squared test, Ho: p2 = p1 versus Ha: p2! = p1

Study parameters: alpha = 0.0500, N = 540, N per group = 270, delta = -0.0590 (difference)

p1 = 0.2410, p2 = 0.1820

Estimated power: = 0.3492

**9. Place of residence, observed OR 1.77**

. power twoproportions 0.12 0.22, test(chi2) n(540) continuity

Estimated power for a two-sample proportions test

Pearson's chi-squared test; Ho: p2 = p1 versus Ha: p2! = p1

Study parameters: alpha = 0.0500, N = 540, N per group = 270, delta = 0.1000 (difference)

p1 = 0.1200, p2 = 0.2200

Estimated power: = 0.8480

**9. Participants level of education, observed OR 1.50**

. power twoproportions 0.515 0.367, test(chi2) n(540) continuity

Estimated power for a two-sample proportions test

Pearson's chi-squared test; Ho: p2 = p1 versus Ha: p2! = p1

Study parameters: alpha =0.0500, N = 540, N per group =270, delta = -0.1480 (difference)

p1 = 0.5150, p2 = 0.3670

Estimated power: = 0.9240

**10. Marital status, observed OR 0.92**

. power twoproportions 0.17 0.107, test(chi2) n(540) continuity

Estimated power for a two-sample proportions test

Pearson's chi-squared test; Ho: p2 = p1 versus Ha: p2! = p1

Study parameters: alpha = 0.0500, N = 540, N per group = 270, delta = -0.0630 (difference)

p1 = 0.1700, p2 = 0.1070

Estimated power: = 0.5139

**11. Fetal birth weight, observed OR 1.95**

. power twoproportions 0.614 0.496, test(chi2) n(540) continuity

Estimated power for a two-sample proportions test

Pearson's chi-squared test; Ho: p2 = p1 versus Ha: p2! = p1

Study parameters: alpha = 0.0500, N = 540, N per group = 270, delta = -0.1180 (difference)

p1 = 0.6140, p2 = 0.4960

Estimated power: = 0.7634

**12. Height of the participant, observed OR 1.08**

. power twoproportions 0.17 0.82, test(chi2) n(540) continuity

Estimated power for a two-sample proportions test

Pearson's chi-squared test; Ho: p2 = p1 versus Ha: p2! = p1

Study parameters: alpha =0.0500, N = 540, N per group = 270, delta = 0.6500 (difference)

p1 = 0.1700, p2 = 0.8200

Estimated power: = 1.0000

**13. Occupation of respondent, observed OR 1.44**

. power twoproportions 0.65 0.61, test(chi2) n(540) continuity

Estimated power for a two-sample proportions test

Pearson's chi-squared test; Ho: p2 = p1 versus Ha: p2! = p1

Study parameters: alpha = 0.0500, N = 540, N per group = 270, delta = -0.0400 (difference)

p1 = 0.6500, p2 = 0.6100

Estimated power: = 0.1397.
