# Supplementary material for: Risk factors for obstructed labour in Eastern Uganda: A case control study
Source: PLoS One. 2020 Feb 10;15(2):e0228856. doi: 10.1371/journal.pone.0228856 (PMC7010384; doi:10.1371/journal.pone.0228856)
Supplement: S3 File — (DOCX) [file pone.0228856.s003.docx]

Response:

Thank you so much for the comment. We agree with the reviewer that most likely we did not have power to detect a difference between the frequency of antenatal care visits (< 4 Vs ≥ 4 ANC visits) and obstructed labour even if the difference had been there*.* This can easily be seen from the confidence intervals that show an imprecise estimate.

We however agree with a huge body of statistical evidence[1, 2] that calculating a post hoc power is logically flawed for the following reasons:

1. Posthoc power is a function of the p value, on a 1:1 relationship (fig 1 from Hoenig et al) and non-significant p values always correspond to low power i.e. we can never reach a conclusion that we failed to reject the null, but since we had high power, there is some evidence that the data supports the null hypothesis. This makes the exercise futile, as it adds nothing to the interpretation of the data.


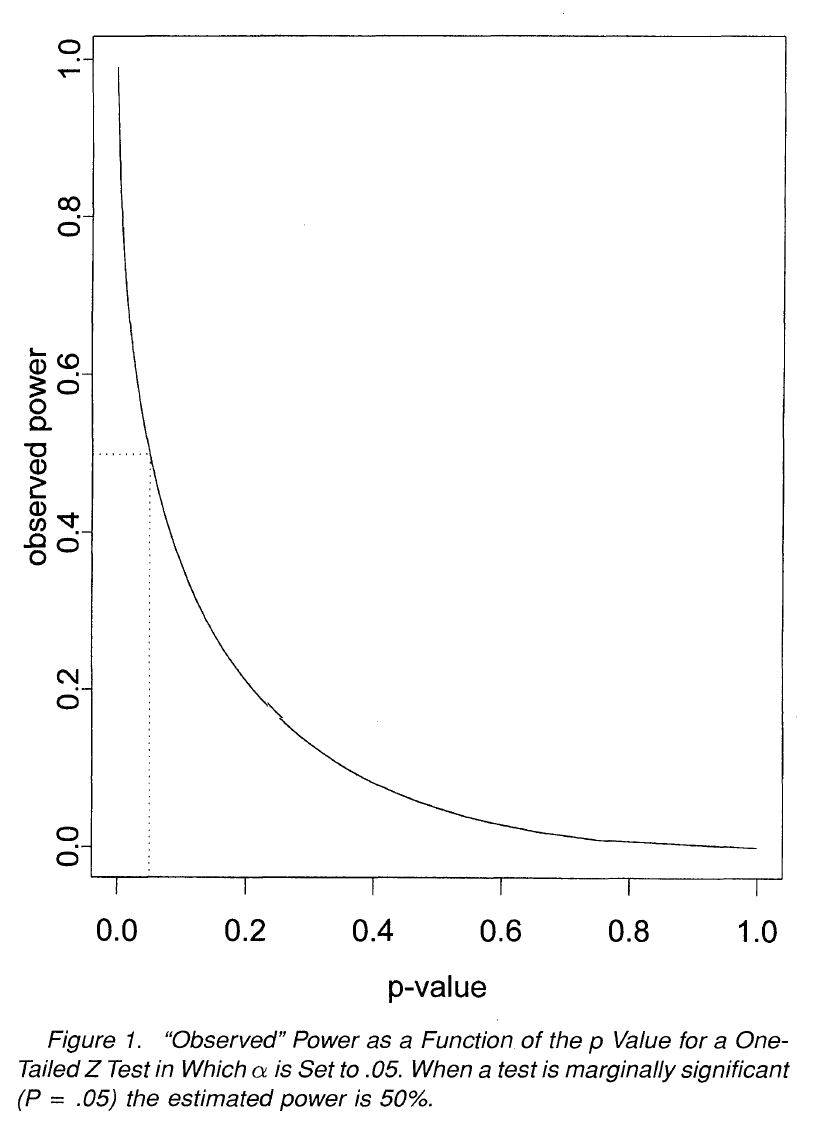


1. An interpretation paradox. Suppose we conduct a posthoc power test on two test results A and B and we discover that posthoc power of A was higher than post hoc power of B; but the p value of A was lower than the p value of B; what shall we conclude. The logic of calculating posthoc power would say that we can conclude that we are more confident in the results of A compared to B in terms of failing to reject the null, but concurrently the interpretation of the p value of A would show stronger evidence against the null compared to the p value of B. What would we do if the interpretation of the post hoc power and p value disagree (which is a statistical possibility)? The obvious answer is that we would stick with the interpretation of the p value; and if that is the case, why do we go ahead with calculating post hoc power if we have results from p values.
2. With the results of a confidence interval, a post hoc power calculation adds no further benefit.
3. Further arguments are presented in the papers referenced.

References

1. Hoenig JM, Heisey DM. The abuse of power: the pervasive fallacy of power calculations for data analysis. The American Statistician. 2001;55(1):19-24.

2. Levine M, Ensom MH. Post hoc power analysis: an idea whose time has passed? Pharmacotherapy. 2001;21(4):405-409.
