# Supplementary material for: Risk factors for obstructed labour in Eastern Uganda: A case control study
Source: PLoS One. 2020 Feb 10;15(2):e0228856. doi: 10.1371/journal.pone.0228856 (PMC7010384; doi:10.1371/journal.pone.0228856)
Supplement: Attachment — Submitted filename: response to the reviewers.docx [file pone.0228856.s004.docx]

20^th^/11/2019

To

Dr. Calistus Wilunda

Academic Editor

PLOS ONE

Dear Dr. Calistus Wilunda

Re; **Response to reviewers’ comments and resubmission of revised manuscript ID PONE-D-19-20983**

Thank you for taking off time to review and provide feedback on this manuscript. After further consideration, we have decided to revise and resubmit it for reassessment. Please receive the revised copy with specific responses and changes summarized in the table below.

| **Reviewers comment** | **Response to comment** | **Line number** |
| --- | --- | --- |
| **Academic editor** | | |
| Old title;  Increased frequency of antenatal care attendance is not protective of obstructed labour in Eastern Uganda: a case control study | New title;  Risk factors for obstructed labour in Eastern Uganda: a case control study | 1 |
| Comment 1;  The study was designed to detect an OR of 2 (which is a quite a huge effect and not based on any evidence) with the resultant sample size being 270 cases. The true effect of ANC on obstructed labour is likely to be too small. | It is possible that the effect of ANC on obstructed labour could be small. However, at the time of designing this study the information at hand to guide our sample size estimation from Ethiopia and Nigeria indicated that the effect of ANC on obstructed labour was as high as 11. So, our choice of an OR of 2 was very conservative since we had no published information from Uganda. We now have results that indicate that the effect of ANC on obstructed labour in Uganda is likely to be low which is in agreement with your observation. Future studies on this subject in Uganda could potentially estimate sample size based on our findings since there seems to be a lot of variations depending on the context. | 112 - 120 |
| Comment 2;  Nonetheless, the authors conclude: "Increased frequency of ANC attendance was not protective against obstructed labour". This is not a statistically valid conclusion because the data are entirely consistent with a strong protective effect. Their study was simply too small to rule out any plausible effect sizes in either direction. | Thank you very much for pointing this out to us, we have revised our conclusion in the write up | 53 – 45  314 – 319 |
| Comment 3;  One more thing to note is that the use of conditional logistic regression is not appropriate for this study design. | Thank you for pointing this out to us, the analysis has been revised appropriately using logistic regression since we did not do any matching in this study. | 44 - 45 160-176 |
| **Reviewer 1** | | |
| Comment 1;  Line #102- Since ACOG appears ones on you document, abbreviation is not recommended. Moreover, give some elaboration about the guideline to make clear for those reader who are far from the discipline. | This has been revised and written out in full | 101 - 102 |
| Comment 2.  Line #109- “Controls were women admitted to the labour suit within the same 24-hour period in active labour without obstruction.” This shows that for every case there must be one control within 24 hours. What if you experience one case but not control within the last hours? Don’t you exclude these women? If you did so, this has to be stated in the exclusion criteria. | Thank you for this observation. However, this did not happen throughout the study because there were many more women without obstructed compared to those with obstructed labour on a daily basis. On average 25 women give birth every day and about 3 of these had obstruction. | NA |
| Comment 3.  Line #126—“Cases were women with OL carrying singleton” why you want to study only on those singleton pregnancy while this variable (singleton Vs Twin ) has to be studied? | Our interest was not to study this variable. Twins are usually compounded with other risks. To avoid compounding of risks, we excluded women with other obstetric emergencies such as antepartum haemorrhage and preeclampsia as either cases or controls | 127 -137 |
| Comment 4.  Line #167-168—“Confounding was considered present if the difference between the crude  and adjusted OR was ≥ 10 percentage points” please put your scientific reference to say so. | The reference has been included | 179 -180 |
| Comment 5.  Line 199- “ Married women were 0.3 times (COR 0.31(0.12 – 0.78) less likely…) This kind of OR reporting has to be modified in your document.  • Eg. If OR=0.31—the odds of OL among married women was 69% less likely than those single mothers.  • If )R=1.31—the odds of OL among married women was 31% more likely to occur than on single mothers.  • But if OR>2- the odds of OL …. Is 2 times…. | Thank you for the observation, after care full consideration we have decided to report the OR’s as odds of outcome in the various risk groups throughout the write up. | 208 – 209  212 – 213  254 – 255  267 – 268  270 – 271 |
| Comment 6. General comments;  • In 2016 guideline, WHO refers to “contact” instead of “visit”. But your document uses “ANC visit”. Therefore, what if you modified it. | Thank you for this observation, we chose to use visit because in Uganda all ANC takes place at the health facilities up to the level of Health Centre at the parish. While in other countries ANC is also carried out using mobile teams/clinics and so they prefer to use the word contact instead of visits. | NA |
| • Since your conclusion shows that the number of ANC visit is no more associated with OL. Don’t you think this can enable you to recommend further research on four ANC (FANC) Vs WHO standard of Eight ANC. | As pointed out by the academic editor, we have revised our conclusions accordingly. However, we strongly agree with your suggestion of further research to inform the change from four ANC visits to the current WHO standard of Eight ANC contacts. Please see the title, abstract and conclusions for details. | 248 – 250 |
| •Post estimation of the final model has to be conducted and presented. | This has been done by checking for the variance inflated factors | 175 - 176 |
| **Reviewer 2;** | | |
| Comment 1.  In the Introduction, line 77, please provide a reference for the statement, “these improvements have not translated into a significant reduction in morbidity and mortality”. | The reference has been provided | 75 - 76 |
| Comment 2.  On line 86, kindly clarify how for “increased frequency of ANC attendance” is defined. Are authors hypothesizing that attending 8 ANC appointment would be protective of OL? | We hypothesised that increased frequency of ANC attendance (<4 versus ≥ 4 visits) was protective of OL. | 85 – 86 |
| Comment 3.  In the Materials and Methods section, study design, line 97, please describe the study design in more detail. | More detail has been provided | 96 - 98 |
| Comment 4.  In Sample size and sampling, line 116-118, explain why authors assumed that women who develop OL are likely to have attended < 4 ANC visits, and the controls were likely to have  attended ≥ 4 ANC visits. | Risk profiling to identify pregnant women at risk of developing adverse maternal and perinatal outcomes is one of the goals of ANC in safe motherhood. Other factors remaining constant, one would think that a pregnant woman who makes more ANC visits is less likely to suffer from adverse outcomes such as OL since she has had more opportunities to be screened for risks. Hence our assumption that cases (women with obstructed labour) are likely to have attended < 4 ANC visits, and the controls were likely to have attended ≥ 4 ANC visits. | 121- 122 |
| Comment 5.  Under Study Variables, authors mention socio-demographic factors highlighted in the literature yet fail to provide citations for factors listed. | The citations have been provided | 144 |
| How did the authors measure having a delivery plan in place and use of herbal medications during labour? | We interviewed the respondents and also cross checked the mother passport (ANC record) to verify if it was documented.  For use of herbal medicines, it was through interviewing the respondents, their next of kin and observation (some of the come in with remnants of the herbal medicines on the body and in the mouth). | 150 – 156 |
| Comment 6.  In Data Analysis, line 164, give information about why significance level of (P<0.25) was chosen. | This was to allow us flexibility in order control for potential residual confounding due to factors that we had not previously hypothesized to be confounders. | 169 - 184. |
| Define for the reader what variables were used in logistic regressions. Mention what covariates were entered in the stepwise CLR model (line 164). Also include all the statistically significant covariates (line 167) used in the final adjusted multivariable model. | This has been revised accordingly in the manuscript. | 179 – 183 |
| Comment 7.  In the Results, how are authors defining short stature (line 178) and normal weight (line 179)? | Short stature < 150 cm and normal birth weight 2.5 to 3.5 Kg | Tables 1,2 and 3. |
| Comment 8.  For Table 1, it would be helpful to expand the number of categories listed for ANC. | Unfortunately, this is not possible at the moment because the categorization was done at the time of data collection. | N/A |
| Was analysis conducted based on whether participants attend less than or greater/equal to 4 ANC?  This is mentioned in the discussion (line 215) but should be mentioned in data analysis section. | Yes  This has been included in the data analysis section | N/A  168 |
| Provide rationale for how this categorization is the most appropriate choice for testing the hypothesis. | Currently, under the focused antenatal care model that is implemented in Uganda, it is recommended that every woman with a normal pregnancy should attend a minimum of four ANC visits for better outcomes. This is what guided our categorization of the ANC visits. | N/A |
| Comment 9.  How did authors adjust for confounding (line 204)? | We adjusted for confounding in the analysis using stratification and multivariable logistic regression. | 163- 175 |
| Comment 10.  In the Discussion, authors neglect to discuss why/how use of herbal medications in labour was associated with OL (line 193). | Thank you for the observation, this has been revised | 252 - 258 |
| Comment 11.  It is not surprising that history of being a referral was risk factor for OL (line 193-196). It would be interesting to know whether OL was the reason for referral. | Most of the women were referred without proper documentation and specific diagnosis of obstructed labour from the referring facilities, but they were diagnosed with obstructed labour on admission. So, it is not surprising that being a referral was a risk factor for obstructed labour | 265- 272 |
| Comment 12.  Describe how the authors adopting an analytical approach versus descriptive explains the contradictory findings (line 253). | This has been clarified | 289 -293 |
| Comment 13  In the conclusion, explain how referral is a potentially modifiable risk factor for OL (line 283). | Strengthening health system at the lower health facilities to reduce delays experienced by women in labour at that level might reduce the number of patients with obstructed labour referred to the regional hospital. | 322 - 324 |
| Comment 14.  Finally, in the References, less than half of the references are from within the last five years. Consider updating references. | We have tried our level best to address this but there are not many current references on this subject. | 347 – 464 |
| Also, there are quite a few incomplete citations in the reference section. | These have been revised and updated accordingly now | 347 – 464 |
| **END** | | |

Sincerely,

Dr. Musaba W. Milton

Department of Obstetrics and Gynaecology

Mbale Regional Referral Hospital/ Busitema University
