# Supplementary material for: Risk factors for obstructed labour in Eastern Uganda: A case control study
Source: PLoS One. 2020 Feb 10;15(2):e0228856. doi: 10.1371/journal.pone.0228856 (PMC7010384; doi:10.1371/journal.pone.0228856)
Supplement: Attachment — Submitted filename: response to the reviewers2.docx [file pone.0228856.s005.docx]

20^th^/01/2020

To

Dr. Calistus Wilunda

Academic Editor

PLOS ONE

Dear Dr. Calistus Wilunda

Re; **Response to reviewers’ comments and resubmission of revised manuscript ID PONE-D-19-20983**

Thank you for taking off time to review and provide feedback on this manuscript. Please receive the revised copy with specific responses and changes summarized in the table below.

| **Reviewers comment** | **Response to comment** | **Line number** |
| --- | --- | --- |
| **Academic editor** | | |
| Please include post hoc power calculations to show the extent to which the study was powered to examine the link between the factors assessed and obstructed labour. This is in the light of the fact that this study was not well powered to assess the link between ANC attendance and the outcome. You can include this information as a supplementary file. | Thank you so much for the comment. We agree with the reviewer that most likely we did not have power to detect a difference between the frequency of antenatal care visits (< 4 Vs ≥ 4 ANC visits) and obstructed labour even if the difference had been there. This can easily be seen from the confidence intervals that show an imprecise estimate. The post hoc power calculations have been done for all the variables studied; referral status, use of local herbs, having a delivery plan, number of ANC visits, prime parity, occupation of the spouse, education level of spouse, distance from the nearest health facility, place of residence, occupation of the spouse, participants level of education, marital status, fetal birth weight and height of respondent.  We however agree with a huge body of statistical evidence that calculating a post hoc power is logically flawed for the reasons highlighted in supplement 3 | Included as a separate file name post hoc power calculations (S1).  Lines 284 to 289, page 17.  Included as a separate file Post hoc power calculations rebuttal (S3) |
| **Journal Requirements:** | | |
| Comment 1;  Please ensure that your manuscript meets PLOS ONE's style requirements, including those for file naming. | We have updated all these accordingly and feel that the manuscript meets PLOS ONE’s style requirements. | NA |
| Comment 2.  We note that you have indicated that data from this study are available upon request. PLOS only allows data to be available upon request if there are legal or ethical restrictions on sharing data publicly.  a) If there are ethical or legal restrictions on sharing a de-identified data set, please explain them in detail (e.g., data contain potentially identifying or sensitive patient information) and who has imposed them (e.g., an ethics committee). Please also provide contact information for a data access committee, ethics committee, or other institutional body to which data requests may be sent.  b) If there are no restrictions, please upload the minimal anonymized data set necessary to replicate your study findings as either Supporting Information files or to a stable, public repository and provide us with the relevant URLs, DOIs, or accession numbers. | Thank you for the guidance, after consultations with the sponsor of the study and the Makerere University School of Medicine Research and Ethics Committee (SOMREC), we have uploaded the minimal anonymized data set necessary to replicate study findings as Supporting Information file (**S2 file. Dataset RFOLMUK**) | NA |
| **Reviewer 1** | | |
| Comment.  In this study, the authors conducted a case-control study to explore the risk factors for obstructed labour (OL) in Eastern Uganda. This study is of great public health importance. Overall, the study was well planned and conducted, analysis was correctly performed, results carefully discussed and manuscript nicely written. The authors also addressed the study limitations. Several issues: | Thank you for the feedback. | NA |
| Issue1;  The full name of an abbreviation should be given at the first appearance. For example, Introduction section, 1st paragraph, Line 63, “PPH”. | Thank you for the observation, this has been written out in full as postpartum hemorrhage. | Line 56, page 4. |
| Issue 2;  Introduction, 2nd paragraph, Line 67, what do you mean by “improving nutrition of the girl child…?” | This has been elaborated further in the manuscript | Lines 61-63, page 4. |
| Issue 3;  Results section, 1st paragraph, Line 199, “159±8.2 Vs 1161.4±7.4”, please check height is correctly presented. | Thank you for the observation, this has been corrected in the manuscript to “159±8.2 Vs 161.4±7.4”, | Line 183, page 9. |
| Issue 4;  The result for the main hypothesis is negative. There is the issue of multiple comparisons, so the conclusions should be drawn with caution. | This limitation has been highlighted in the discussion section | Line 233 to 236, page 15 |
| Issue 5;  The presentations of Table 2 and Table 3 should be improved. The reference category usually should be consistent, either at the top or at the bottom. | All the tables have been reviewed and revised | Table 1: Line 185 to186, page 9 to 11  Table2: Line 200 to201, page 11 to 12  Table 3: Line 209 to 210, page 13 to 14. |
| Issue 6;  Typos and grammatical errors need to be checked and corrected. | These have been checked and corrected throughout the document | NA |
| **Reviewer 2;** | | |
| Comment 1.  While the study presents the results of original research, the statistics and other analyses still need to be described in more sufficient detail to know whether they were performed to a high technical standard. The conclusions need to be presented in a more robust fashion and provide more explanation of what the data support and results show. | The statistical analysis has been described in more detail and the data set is attached.  We have also added a post hoc power calculation.  Discussed the power limitations in more detail | Line 150 to 171, page 8 and 9.  Supplement file S1.  Lines 284 to 289, page 17. |
| Comment 2.  Even though the article is presented is written in standard English, it could be improved with additional copy-editing for correct grammar usage. | This has been done throughout the document | NA |
| Comment 3.  Revisions would be easier to track if authors had highlighted or underlined changes in the text | Sorry for the inconvenience, we have included a revised copy of the manuscript with track changes. | Throughout the document. |
| **END** | | |

Sincerely,

Dr. Musaba W. Milton

Department of Obstetrics and Gynaecology

Mbale Regional Referral Hospital/ Busitema University
